# Clinical experience of comprehensive treatment on the balance function of Parkinson's disease

**DOI:** 10.1097/MD.0000000000020154

**Published:** 2020-05-08

**Authors:** Haitao Li, Siquan Liang, Yang Yu, Yue Wang, Yuanyuan Cheng, Hechao Yang, Xiaoguang Tong

**Affiliations:** a Graduate School of Tianjin Medical University; b Department of Neurosurgery, Tianjin Huanhu Hosptial; c Department of Neurological Rehabilitation, Tianjin Huanhu Hospital; d Department of Psychology, Tianjin Huanhu Hosptial, Tianjin, China.

**Keywords:** balance dysfunction, deep brain stimulation, medication, Parkinson's disease, psychotherapy, rehabilitation, subthalamic nucleus

## Abstract

To investigate the effect of multi-disciplinary teamwork on balance performance of Parkinson's disease (PD).

Sixteen primary Parkinson's disease patients (8 male, 8 female) treated with bilateral subthalamic nucleus deep brain stimulation (STN-DBS) were included in the study. The median age of patients was 60.5 years; all patients were in the Hoehn&Yahr (H&Y) 3 stage; the median PD duration of the disease was 9 years. For each patient, multi-disciplinary teamwork treatment including DBS, medication, physical therapy and psychotherapy proceeded. levodopa equivalent daily dose (LEDD, mg/day), life quality (PDQ-39), Motor disability (MDS-UPDRSIII) and balance performance (MDS-UPDRS 3.12, Berg Balance Scale BBS, Limits of Stability LoS) were assessed in different time and status respectively: preoperation (Med-off, Med-on), postoperation (Stim-Off/Med-Off, Stim-On/Med-Off, Stim-On/Med-On), 6 months postoperation (Stim-On/ Med-Off, Stim-On/Med-On) and 12 months postoperation (Stim-On/Med-Off, Stim-On/Med-On).

The LEDD, life quality (PDQ-39) continued to improve during the follow-up, statistical difference were found in both 6 months postoperation and 12 months postoperation compared with preoperation. The Motor disability (MDS-UPDRSIII), balance performance (MDS-UPDRS 3.12, BBS) and the LoS (target acquisition percentage, trunk swing angle standard deviation, time) showed significant improvement in Stim-On/med-Off 6 months postoperation and 12 months postoperation separately compared with Med-Off preoperation.

Multi-disciplinary teamwork for PD patients with STN-DBS could improve balance performance.

## 1 Introduction

Primary Parkinson's disease (PD) is a common neurodegenerative disease in the elderly, with symptoms such as bradykinesia, tremor, rigidity, and balance dysfunction. Balanced dysfunction causes PD to increase body swing, posture instability, decreased coordination, and becomes more and more serious as PD progresses, and has been identified as one of the independent risk factors for falls. About 45% to 68% of PD patients fall every year, and about two-thirds of them experience more than one fall.^[1]^ Falling can bring a series of serious consequences, such as hip fractures, brain injuries, etc. Because of the fear of falling, the activities of patients with PD are restricted, which seriously affects the quality life of patients, even life safety.^[2]^ In this study, we investigate the effect of multi-disciplinary teamwork on balance performance in PD patients with bilateral STN-DBS.

## 2 Methods

### 2.1 Subjects

Twenty Primary PD patients were treated with bilateral STN-DBS in Tianjin Huanhu Hospital from December 2017 to August 2019, 2 patients were lost to follow-up and 2 patients had deficient clinical material. Sixteen PD patients were included after all (8 male, 8 female), the median age of patients was 60.5 year (Q25∼Q75 = 57.25∼64.75). All the patients were in the H&Y 3 stage, with a median PD duration of 9 years (Q25∼Q75 = 7∼13.5). Include criteria:

1. Primary PD confirmed by 2015 MDS diagnostic criteria;
2. MDS-UPDRS III score improved greater than 30% in acute levodopa drug test;
3. Qualified to surgical treatment, underwent bilateral STN-DBS.^[3]^

Exclusion criteria:

1. Balance dysfunction caused by secondary Parkinson's syndrome, hydrocephalus, intracranial tumors, multiple cerebral infarctions, etc;
2. Patients with obvious cognitive dysfunction;
3. Serious anxiety, depression, and mental illness.

All patients signed informed consent.

### 2.2 Ethical approval

The study was approved by the Medical Ethics Committee of Tianjin Huanhu Hospital (No. 2019-35) and was conducted according to the Declaration of Helsinki principles.

### 2.3 Clinical assessment

Mean LEDD of patients preoperation was 1225.63 ± 714.81 mg. For each patient, multi-disciplinary teamwork treatment including DBS program, medication, rehabilitation, and psychotherapy was preceded.

#### 2.3.1 DBS surgery procedure

All surgery was taken by the same surgical team. All patients underwent bilateral STN-DBS. Leksell Stereotactic System (Elekta, Stockholm, Sweden) and the Frame Link planning system (Medtronic, Minneapolis, MN) were used for preparation of surgery. According to the Schaltenbrand-Wahren atlas, the tentative target site was 2 mm posterior to the midpoint of the anterior–posterior commissure (AC-PC) line, 12 mm lateral to the AC-PC line, and 4 mm ventral to the AC-PC line. Target sites were corrected based on T2-weighted MRIs. The target was reconfirmed physiologically with an intraoperative microelectrode recording, just prior to performing the test stimulation studies. After all, quadripolar DBS electrodes (Activa 3389s, Medtronic) were implanted bilaterally in stereotactic guide.

After general anesthesia induced, implantable pulse generators (Activa RC Medtronic) were implanted subcutaneously in the subclavian pockets of the chest wall and connected to the DBS leads subcutaneously. Imagines of computed tomography postoperative and MRI preoperative were superimposing in Frame Link planning system to insure the local accuracy of electrode placement.

#### 2.3.2 DBS programming

All patients were switched on at 4 weeks after the operation (Med-Off). During programming, the limb with severe symptoms is programmed prior. Test the corresponding contactors one by one according to the patient's symptoms, with attention of the symptom control and drug side effects. In many of stimulation mode, monopolar stimulation is the most utilized, and interleaved stimulation is applied in patients with complicated symptom. Ways to solve the problems in balance performance include: down-regulate the stimulation voltage; change the electrode contacts, try the contacts in substantia nigra reticulum; decrease the frequency and interleaved stimulation. Adjust the stimulation parameters, electrode contacts, and drugs dose to optimize from 3 to 6 months after the surgery. The purpose is to alleviate the symptoms and prevent drug side effects. In principle, the best clinical symptom improvement is obtained with the minimum stimulation intensity.

#### 2.3.3 Medication

The first time drug dose postoperation is the same as preoperation; the principle of medication is to effectively improve symptoms and life quality; adhere to “dose titration” to avoid the acute side effects of drugs, and achieve "satisfactory” clinical results in minimum doses. With motor control by DBS, reduce the dose of levodopa and dopamine agonists gradually. In patients with freezing of gait, down-regulates the stimulation and increase the compound levodopa dose at the same time. Pay attention to maintaining the symmetry of muscle tension of both limbs while taking medication, taking into account factors such as orthostatic hypotension. Drug dose was adjusted to be stable from 3 to 6 months after surgery.

#### 2.3.4 Rehabilitation

Rehabilitation training for PD patients mainly includes center of gravity shift, posture stability, and sensory function. The training of transfer gravity center is the training of the core strength. Through the bridge movement, turning over training and other methods to increase the core strength of patients in sitting position - kneeling position (4 points - 3 points - 2 points support) - standing position. Postural stability training includes squat strategy, hip strategy, and stepping strategy to enhance the patient's application of the balance strategy. The training of sensory function requires train the patient's proprioception on cushion, balance board, combined with the balance strategies above. In addition, rehabilitation training includes training in all directions, turning training and so on.

#### 2.3.5 Psychotherapy

The prevalence of psychiatric symptoms in PD patients is high, and for this part of the patients, it is first identified whether it is caused by anti-Parkinson drugs or the disease itself. If it is induced by anti-Parkinson drugs, it could be reduced or discontinued according to the probability of inducing mental disorders. If the effect of adjusting the drug is not ideal, it suggests that the mental disorder may be caused by PD; use clozapine or quetiapine for hallucinations and delusions; use selective serotonin reuptake inhibitors or dopamine receptor agonist for depression and anxiety, active palpitations; lauraxi and diazepam relieve irritability; cholinesterase inhibitors treat patients with cognitive impairment and dementia.^[4]^

Motor disability (MDS-UPDRS III) and balance performance (MDS-UPDRS 3.12, BBS, LOS) were assessed in different time and status respectively: preoperation (Med-off, Med-on), postoperation (Stim-Off/Med-Off, Stim-On/ Med-Off, Stim-On/ Med-On), 6 months post-operation (Stim-On/ Med-Off, Stim-On/ Med-On) and 12 months postoperation (Stim-On/Med-Off, Stim-On/Med-On). Before the assessment, the patients were stopped with dopamine agonist for 72 hours, compound levodopa and other anti-PD drugs for 12 hours. The quality of life during follow-up was assessed by PDQ-39.

The limit of stability (LOS) is the maximum displacement of the body mass center (COM) in all directions while standing, without falling or striding. When the TecnoBody PROKIN instrument detects LOS, the patient can visually look at the front display. The feet are flush with the shoulders, standing on the balance board, and the feet are not moving, keeping the body straight. At the beginning of the test, the screen will display the target points in eight main directions (front, back, left, right, left front, right front, left rear, right rear) in turn, and the subject tilts the body quickly and accurately from the center to the target direction. The movement body mass is maximally possible to make the display indicator icon close to the target point and record the percentage of the patient's target reaching, the target body swing angle standard deviation and time.

During the test, the subject is not allowed to speak, not allowed to look around, not allowed to move small limbs and other small movements; and tell the subject in advance what action is needed. After the instructing doctor to issue relevant instructions, the subject performs the relevant action, and during this period, if there is an action that does not meet the requirements, the specified action needs to be restarted.

### 2.4 Statistical methods

Statistical analysis was performed using SPSS 19.0 software. Nonparametric statistics were used. Median, the first and third quartiles (Q25–Q75) are used to describe group results. The paired *t* test is used for data of PD at two similar time points. *P* < .05 was considered statistically significant.

## 3 Results

The average LEDD at 6 months postoperation (503.13 ± 140.50 mg) and 12 months postoperation (464.06 ± 139.03 mg) were significantly decreased during the follow-up (*P* < .05). The PDQ-39 at 6 months postoperation (44.63 ± 22.38) and 12 months postoperation (38.56 ± 25.34) were improved obviously than preoperation (81.88 ± 27.97) (*P* < .05) (Table 1).

**Table 1:**
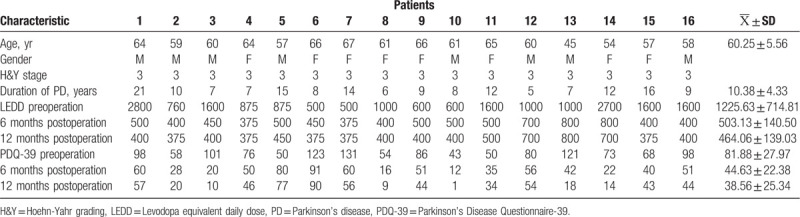
Clinical material of 16 PD patients.

The Motor disability (MDS-UPDRSIII) is higher in Med-On preoperation than Med-Off preoperation. The balance performance (MDS-UPDRS 3.12 and BBS) had no differences at Med-On preoperation than Med-Off preoperation. During the follow-up, the MDS-UPDRS III, MDS-UPDRS 3.12 and BBS were significantly improved in the state of Stim-On/Med-Off postoperation compared with the state of Stim-Off/Med-Off postoperation and Med-Off preoperation. It continued to improve during the follow-up. Statistical difference were found in the state of 6 months postoperation Stim-On/Med-Off and the state of 12 months postoperation Stim-On/Med-Off seperately compared with the former test. No differences were found between Med-On and Med-Off in the state of Stim-On (Table 2).

**Table 2:**
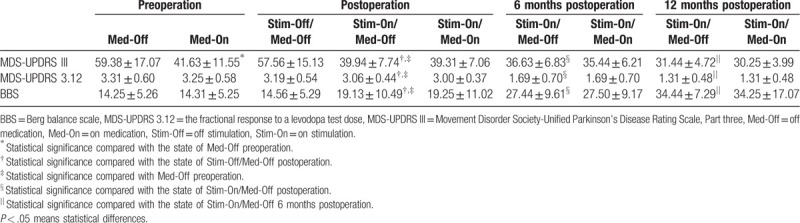
Motor disability (MDS-UPDRS III) and balance performance (MDS-UPDRS 3.12, BBS) during the follow-up.

During the follow-up, the LOS (Target acquisition percentage, swing angle standard deviation and Time) were significantly improved in the state of Stim-On/Med-Off postoperation compared with the state of Stim-Off/Med-Off postoperation and Med-Off preoperation separately, while no difference was found between Stim-On/Med-Off postoperation and Med-On preoperation. They continued to improve during the follow-up. Statistical difference was found in the state of 6 months postoperation Stim-On/Med-Off and 12 months postoperation Stim-On/Med-Off seperately compared with Stim-On/Med-Off postoperation. No differences were found between Med-On and Med-Off in the state of Stim-On (Table 3).

**Table 3:**
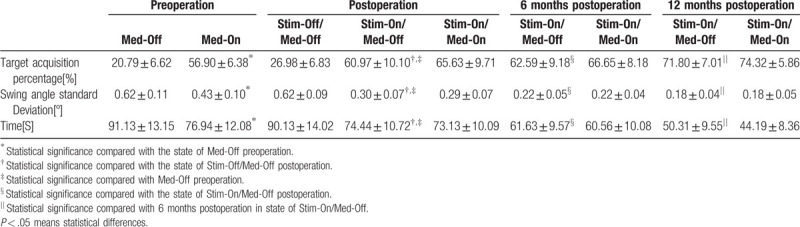
Evaluation of LOS during the follow-up.

**Figure 1:**
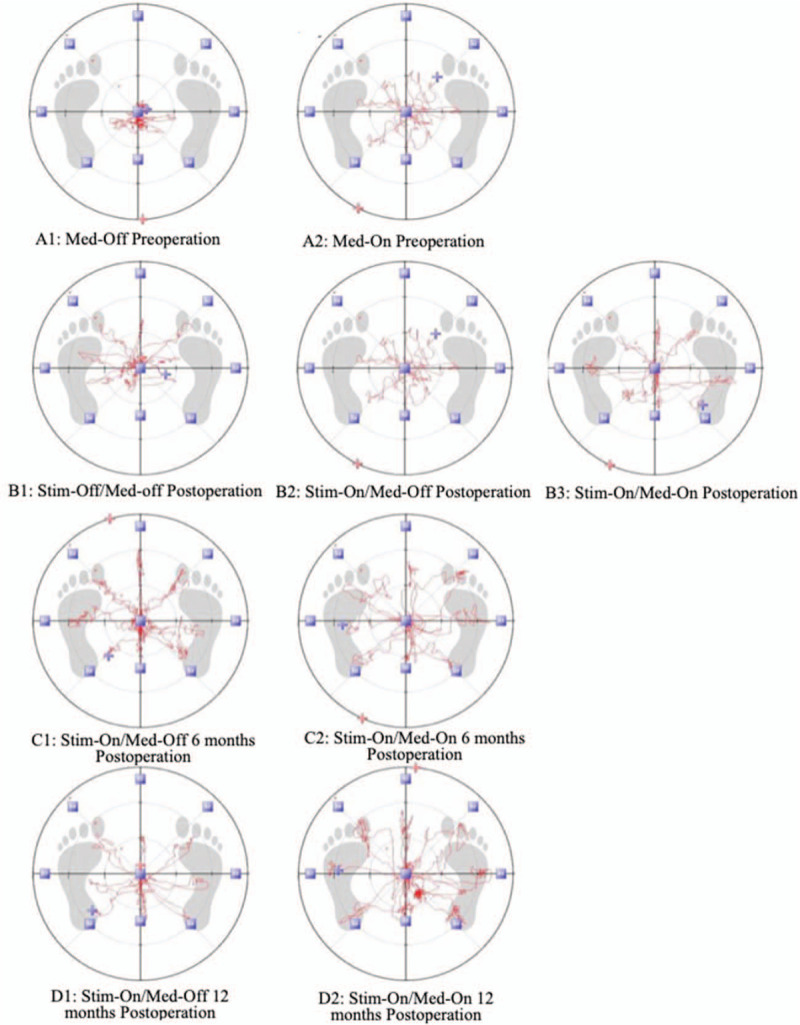
The Target acquisition percentage postoperation in different states (B2, B3, C1, C2, D1, D2) are obviously better than Med-Off preoperation (A1). It significantly improved in the state of Stim-On/Med-Off postoperation compared with the state of Stim-Off/Med-Off postoperation and Med-Off preoperation separately, while no difference was found between Stim-On/Med-Off postoperation and Med-On preoperation. They continued to improve during the follow-up. Statistical difference were found in the state of 6 months postoperation Stim-On/Med-Off and 12 months postoperation Stim-On/Med-Off seperately compared with Stim-On/Med-Off postoperation. During the follow up, the Target acquisition percentage of Stim-On/Med-On had no differences with Stim-On/Med-Off at the same time. The red lines are movement orbit of the center of gravity when patients were asked to tilts the body quickly and accurately from the center to the target direction (front, back, left, right, left front, right front, left rear, right rear) in turn.

The target acquisition percentage postoperation in different states (Fig. 1: B2, B3, C1, C2, D1, D2) are obviously better than med-off preoperation (Fig. 1A1). It significantly improved in the state of Stim-On/Med-Off postoperation compared with the state of Stim-Off/Med-Off postoperation and Med-Off preoperation separately, while no difference was found between Stim-On/Med-Off postoperation and Med-On preoperation. They continued to improve during the follow-up. Statistical difference was found in the state of 6 months postoperation Stim-On/Med-Off and 12 months postoperation Stim-On/Med-Off separately compared with Stim-On/Med-Off postoperation. During the follow up, the Target acquisition percentage of Stim-On/Med-On had no differences with Stim-On/Med-Off at the same time.

## 4 Discussion

Factors affecting the balance function of primary PD are complicated: Decreased dopamine level as a result of dopaminergic neurons loss in substantia nigra leads to motor dysfunction such as bradykinesia, rigidity, and tremor; reduced activity of the cholinergic system by loss of cholinergic neurons in pedunculopontine nucleus and basal forebrain BF could also damage the balance function. Abnormal activity in the cortex, STN, brainstem reticular formation, mesencephalic locomotor region and substantia nigra reticulum (SNr) could cause dysfunction in motor control, sensory processing, cognition or behavior. Especially there are extensive links between pedunculopontine nucleus and SNr, basal ganglia system, motor area, premotor area, supplementary motor area (SMA) and visual area of frontal lobes. Any abnormality could cause obstacles in posture dystonia, etc. Cerebellum lesion especially the cerebellar vermis could cause abnormal motor patterns and balance dysfunction.^[5]^

In addition, functional MRI show vestibular, visual, proprioceptive cortex, motor area, premotor area, SMA, intermediate cingulate cortex, medial and ventral lateral thalamus, globus pallidus, putamen, striatum, pontine, motor area of midbrain and cerebellar vermis are involved in the adjustment of balance, any abnormalities would affect the balance function.^[6,7]^ Although it is difficult to specify the pathological mechanism according to symptoms of balance disorder due to the complexity cause of balance dysfunction, multi-disciplinary teamwork (DBS program, medication, rehabilitation, and psychology) of PD patients with STN-DBS has been proved effective to improve the life quality and balance performance in this study.^[8]^

Although no differences were found between Med-On and Med-Off in the state of Stim-On, our patients showed obviously improve in Med-on preoperation compared with Med-off preoperation, it suggests that drug effect might be concentration-dependent. Dopamine replacement improves PD motor symptom such as bradykinesia, rigidity due to dopamine deficiency in the substantia nigra. Drug therapy could also improve anticipatory postural adjustment, compensatory postural adjustment, and cognition in PD, which could affect the balance performance indirectly. However, the direct effects of drugs on balance performance were variously reported.^[9,10]^ It might be due to the complexity cause of balance dysfunction, drugs might be multiple effects in a different area.

During the follow up, balance performance and LOS in Stim-On/Med-Off postoperation improved significantly compared with Med-Off preoperation and Stim-Off/Med-Off postoperation, which suggests that STN-DBS could improve the balance performance and LOS. STN-DBS may improve the central integration of sensory feedback and conflict by regulating the activity of neurons such as STN and oscillation patterns. In the entire cortical-striatum-pallid-thalamic-cortical system loop, STN-DBS could improve the sensory-motor strategy system, enable the body to better respond to external stimuli and maintain balance.^[11]^ In addition to improving the main motor symptoms of PD, STN-DBS can also improve medical complications such as dyskinesia and symptom fluctuations, which is helpful for improving balance performance. At the same time, group of researchers found that DBS could even worsen the balance function in PD.^[12]^ In our opinion, it might be relate to patients’ subtype, balance performance preoperation, PD stage and management postoperation.

The patients’ balance performance continued to improve from 6 months to 12 months postoperation. We thought that physical therapy plays an important role. Studies have shown that specialized torso physical therapy, including active self-correction training with visual and proprioceptive feedback, passive and active torso stabilization exercises and functional exercises, can significantly improve the patient's balance performance.^[13]^ Treadmill interference training can significantly improve the motor symptoms of PD patients continual, especially in terms of gait and posture balance.^[14]^ Directional balance rehabilitation training can significantly improve patients’ balance control ability and balance performance than simple resistance rehabilitation training.^[15]^ Based on the patient's motor and balance dysfunction data observed through Tecnobody PROKIN balance system, we made rehabilitation personally. In our study, rehabilitation training plays an important role in the continual improvement of patients’ motor function from 6 months to 12 months postoperation, with steady medical dose and stimulates frequency. Long duration of disease (16 patients in this study had a median duration of 9 years range from 5 to 21 years) results in dystonia of torso and limb and abnormal posture; Bradykinesia make it harder for the conversion of body; Decreased response leads to unstable posture; Short stride and poor coordination of limbs results in balance dysfunction. Even more, lack of professional train leads to compensatory or even wrong movement modes. In this condition, professional physical therapy plays a vital role in the treatment of PD motor disability. Physical therapy could correct the wrong movement modes and strengthen the balance strategies to improve the balance performance.^[16,17]^ More attention should be given to physical therapy in clinical practice.^[18]^

Anxiety and depression are the main psychological problems in PD patients. The prevalence of depression is 50% to 70%, and the prevalence of anxiety is 25% to 45%. In patients with anxiety and depression coexists, life quality and medical effective would be seriously affected. Medications including selective serotonin reuptake inhibitors and dopamine receptor agonists play an important role in this regard. At the same time, cognitive-behavioral therapy in PD is gaining more attention. Cognitive-behavioral therapy is a psychological treatment method for mental illnesses such as depression and anxiety. It emphasizes the improvement of cognition, which in turn produces emotional and behavioral changes, and plays an important role in the treatment of PD.^[19,20]^ In our study, psychological health of all 16 patients had been evaluated. During the treatment, 6 with anxiety and 8 with depression improved with psychotherapy.

Controversial effects of DBS on PD balance performance in different study result from lack of objective criteria to evaluate the balance function.^[21,22]^ In our study, TecnoBody PROKIN was used to evaluate balance function. TecnoBody PROKIN Balance System is a sensor network that can perform various angular motions on a fine tilt plate. When a patient stands on the plate, the balance data collection board converts each motion into an electrical pulse. All the data is transmitted to the computer system and processed. In this way, balance characteristics such as the LOS can be objectively evaluated (Fig. 1), the balance function of patients could be seen unaided.

In this paper, PD patients stay still, and then change the body gravity to move the screen indication point close to the target, to obtain the target activity percentage and other parameters. This process needs to be located in axial posture, with the joint of hip, knee, and ankle and muscles involved. PD patients with balance disorders may have abnormal parameters due to defects in the sensory and the motor system under the control of the central nervous system.

During the follow up, MDS-UPDRS 3.12 and BBS in Med-On preoperation had no improvement compared with Med-Off preoperation. During the follow up postoperation, MDS-UPDRS 3.12 and BBS improvement was observed. While the TecnoBody PROKIN's assessment of patient stability was found significantly improved between Med-Off preoperation and Med-On preoperation immediately and it continued to improve during follow-up. Here we believe that the TecnoBody PROKIN Balance Test System is more sensitive to assessing patient balance stability than traditional balance questionnaires.

## 5 Conclusions

The complexity and multi-factor of the balance dysfunction in PD patients made it harder to study balance function. In this study, the TecnoBody PROKIN system was used to quantitative analyze the balance data objectively. It is helpful to analyze the balance function and guide the treatment. As a widely used target in DBS surgery, STN is effective and easy to manage. STN-DBS could reduce the drug dose and the economic burden of patients. At the same time, supplemented with drugs, rehabilitation, and psychotherapy could strengthen the clinical improvement.^[8]^^.^ However, control study and continual follow-up need to be further studied in the future to observe the pathological process of balance function in PD patient.

## Author contributions

**Conceptualization:** Siquan Liang, Yang Yu, Xiaoguang Tong.

**Data curation:** Haitao Li, Yue Wang, Yuanyuan Cheng, Hechao Yang.

**Formal analysis:** Haitao Li, Siquan Liang, Yang Yu.

**Investigation:** Haitao Li, Siquan Liang, Yang Yu, Yue Wang, Yuanyuan Cheng, Hechao Yang, Xiaoguang Tong.

**Methodology:** Siquan Liang, Yang Yu, Haitao Li, Xiaoguang Tong.

**Supervision:** Siquan Liang, Yang Yu, Xiaoguang Tong.

**Validation:** Haitao Li, Siquan Liang, Yang Yu, Hechao Yang, Xiaoguang Tong.

**Writing – original draft:** Haitao Li, Siquan Liang.

**Writing – review & editing:** Haitao Li, Siquan Liang, Xiaoguang Tong.
